# Clinical and Evolutionary Implications of Dynamic Coagulotoxicity Divergences in *Bothrops* (Lancehead Pit Viper) Venoms

**DOI:** 10.3390/toxins14050297

**Published:** 2022-04-22

**Authors:** Lachlan Allan Bourke, Christina N. Zdenek, Anita Mitico Tanaka-Azevedo, Giovanni Perez Machado Silveira, Sávio Stefanini Sant’Anna, Kathleen Fernandes Grego, Caroline Fabri Bittencourt Rodrigues, Bryan Grieg Fry

**Affiliations:** 1 Venom Evolution Lab, School of Biological Sciences, University of Queensland, St. Lucia, QLD 4072, Australia; christinazdenek@gmail.com; 2 Laboratrio de Herpetologia, Instituto Butantan, Sao Paulo 05503-900, Brazil; anita.azevedo@butantan.gov.br (A.M.T.-A.); giovanni.silveira@butantan.gov.br (G.P.M.S.); savio.santanna@butantan.gov.br (S.S.S.); kathleen.grego@butantan.gov.br (K.F.G.); cfabri3@gmail.com (C.F.B.R.)

**Keywords:** *Bothrops*, venom evolution, coagulopathy, procoagulant, anticoagulant, fibrinogen

## Abstract

Despite coagulotoxicity being a primary weapon for prey capture by *Bothrops* species (lancehead pit vipers) and coagulopathy being a major lethal clinical effect, a genus-wide comparison has not been undertaken. To fill this knowledge gap, we used thromboelastography to compare 37 venoms, from across the full range of geography, taxonomy, and ecology, for their action upon whole plasma and isolated fibrinogen. Potent procoagulant toxicity was shown to be the main venom effect of most of the species tested. However, the most basal species (*B. pictus*) was strongly anticoagulant; this is consistent with procoagulant toxicity being a novel trait that evolved within *Bothrops* subsequent to their split from anticoagulant American pit vipers. Intriguingly, two of the arboreal species studied (*B. bilineatus* and *B. taeniatus*) lacked procoagulant venom, suggesting differential evolutionary selection pressures. Notably, some terrestrial species have secondarily lost the procoagulant venom trait: the Mogi Mirim, Brazil locality of *B. alternatus*; San Andres, Mexico locality of *B. asper*; *B. diporus*; and the São Roque of *B. jararaca*. Direct action on fibrinogen was extremely variable; this is consistent with previous hypotheses regarding it being evolutionary decoupled due to procoagulant toxicity being the primary prey-capture weapon. However, human patients live long enough for fibrinogen depletion to be clinically significant. The extreme variability may be reflective of antivenom variability, with these results thereby providing a foundation for such future work of clinical relevance. Similarly, the venom diversification trends relative to ecological niche will also be useful for integration with natural history data, to reconstruct the evolutionary pressures shaping the venoms of these fascinating snakes.

## 1. Introduction

Snake venoms exert potent pathophysiological effects upon any physiological pathway reachable by the bloodstream, and with blood coagulation itself potently affected by many venomous snake species [1,2,3,4,5,6]. Venoms may be procoagulant via the activation of blood clotting factors (e.g., factor X activators and prothrombin activators) [1,2,4,5,6] or anticoagulant via the inhibition of factors (e.g., factor IX and X inhibitors), the hydrolysis of phospholipids, or the cleaving of factors in a destructive, non-clotting manner [3,6,7,8]. Fibrinogen is a common zymogen that is targeted by venoms. It may be destructively cleaved by metalloproteases or serine proteases in a non-clotting manner, thereby contributing to anticoagulation through the depletion of fibrinogen levels [8]. Another direct venom action upon fibrinogen is pseudo-procoagulation (a form of anticoagulation), whereby kallikrein-type serine proteases (aka ‘thrombin-like’) cleave fibrinogen imprecisely, leading to a disordered-lattice network that culminates in weak clots that quickly break down, thereby also contributing to the depletion of fibrinogen levels [1,3,4,5,6,9]. 

Despite their medical significance, most groups of venomous snakes have not been investigated in a systematic manner. The wide-ranging genus *Bothrops* (lancehead pit vipers) is no exception. *Bothrops* is a highly speciose genus of pit viper, with 45 species currently recognised in the genus [10], distributed across Latin-America (Mexico, Central America, and South America). The speciose nature and wide range of *Bothrops* across Latin America is a result of its evolutionary history. The common ancestor of the *Bothrops* genus was the first viper to invade South America approximately 23–10 million years ago [11] and it rapidly diversified into the numerous vacant niches, devoid of vipers, across the landscape. Today, *Bothrops* species are found across many habitats and diverse niches [11,12,13,14], allowing for both inter- and intra-specific venom variation. In fact, *Bothrops* are known to exhibit geographical venom variation, with the same species having different venom action based on their geography/habitat [15,16,17]. For example, Segura et al. [17] found venom variation between Mexican and Costa Rican *B. asper* venom samples, with Mexican *B. asper* venom having higher lethality to mice and higher in vivo defibrinogenating activity, but lower in vivo haemorrhagic activity and lower in vitro coagulant activity compared to Costa Rican *B. asper* venom samples. Sousa et al. [16] investigated, at a finer scale, *B. atrox* in a section of the Brazilian Amazon (western region of the state of Pará), and found differences in venom composition and in the function between venom samples collected from different habitats. Consequently, due to the large and intricate landmass of Latin America and the many habitat types one species may inhabit across its range, more studies on intra-specific, geography-based venom variation are needed in this genus.

Each year in Latin America, there are approximately 137,000–150,000 cases of envenomation from snakes and 3400–5000 deaths [18]. *Bothrops* accounts for 70–96.6% of enevenomations in South America [19,20,21,22]. Bites are commonly from *B. atrox* and *B. asper* [22,23,24], although other species are also involved in the snakebite burden on both the mainland (e.g., *B. jararaca* [25], *B. jararacussu* [26], *B. pictus* [27]) and on the Caribbean islands (e.g., *Bothrops lanceolatus* on Martinique and *B. caribbaeus* on Saint Lucia [28,29,30,31]). In addition to bites by the aforementioned terrestrial species, bites also occur from the highly derived arboreal species *B. bilineatus* and *B. taeniatus* [32]. In central America 50–80% of bites are attributed to *Bothrops* (specifically *B. asper*) [24,33]. Mexican epidemiological data are scarce, with little information on the snakes responsible for envenomations, although *B. asper* possibly causes a large number of envenomations [34]. It is estimated there are approximately 28,000 envenomations per year in Mexico [35].

*Bothrops* species are well known for their coagulotoxic venom effects, with procoagulant venom action on human plasma often found in venom effect studies [15,36,37] Amongst other actions, prey capture by *Bothrops* species is facilitated by potent activation of factor X and prothrombin by metalloprotease enzymes; this causes an overdose scenario in which massive amounts of endogenous thrombin are produced, leading to rampant generation of well-ordered fibrin clots [36,38,39,40,41,42,43]. This ultimately leads to prey subjugation through stroke induction [41,44,45]. Pseudo-procoagulant activity—whereby thrombin is cleaved imprecisely, producing weak, transient clots that are easily broken down—has also been observed in *Bothrops* venoms when tested on human fibrinogen [43,46]. In fact, kallikrein-type serine protease enzymes responsible for pseudo-procoagulant activity are common among *Bothrops* venoms [47,48,49,50,51]. As this action is slower than the procoagulant toxicity, it has been proposed as being an evolutionary relic left over from the anticoagulant snakes from which *Bothrops* diverged prior to uniquely evolving procoagulant toxicity [36,43]. However, while the prey capture role is unresolved, the direct action upon fibrinogen is of relevance to human envenomations, as bite victims survive long enough for fibrinogen depletion to become a significant variable, and thereby contribute to consumptive coagulopathy [24,52]. Other activities of clinical significance have been documented in *Bothrops* venoms, including direct actions upon platelets, and vessel damage [53]. 

Prior studies have demonstrated that potent procoagulant toxicity through the activation of both factor X and prothrombin is a defining trait of *Bothrops* venoms [42,43,46]. This distinguishes them from other members of the American pit viper clade, which are typically anticoagulant (including pseudo-procoagulant) in their action upon the blood [37,54,55,56]. A seminal study on this subject revealed that of the 26 *Bothrops* species studied, only *B. taeniatus* (formerly *B. castelnaudi*) was anticoagulant [46]. Notably, this species is also highly derived in that it is one of the few species that is fully arboreal. Inhibition of coagulation factors was noted as the anticoagulant action [46,57]. Furthermore, in Nahas et al. [46] the lack of a direct pseudo-procoagulant action upon fibrinogen was noted for *B. erythromelas* which, instead, destructively cleaved fibrinogen in a non-clotting manner. In a recent study it was shown that *B. erythromelas* inability to clot fibrinogen is due to the deletion of SVTLE genes, which are responsible for fibrinogen clotting [58]. 

A common haemostatic assay used in clinical settings to manage and treat patients and predict patient outcomes is thromboelastography (TEG) [59,60,61,62]. TEG is extremely useful for measuring blood coagulation parameters as a myriad of aspects are ascertained simultaneously, including amplitude (A) (strength of the clot) and reaction time (R) (time until clot formation), thus aiding in the diagnosis and treatment of patients admitted to hospital with coagulopathy. In fact, it has proven useful in guiding the management of coagulopathy in snakebite patients [63]. This technology has also gained traction in the scientific research world, in which researchers use it to test the coagulotoxic action of diverse snake venoms on fibrinogen and plasma, including *Bothrops* species [9,36,37,64,65]. 

Despite their evolutionary novelty and extreme medical importance, the relative effects of *Bothrops* venoms upon coagulation are data-deficient. While an extensive amount of research on *Bothrops* species and their venom (specifically *B. atrox and B. asper*) has been undertaken, many species’ coagulotoxic venom action remains relatively under-studied, including the arboreal *B. bilineatus* and *B. taeniatus*, and island dwelling *B. caribbaeus*. To date, a single paper examines the in vitro effects of *B. caribbaeus* venom on human plasma [66]. Only a few studies on the in vitro coagulotoxic effects of *B. bilineatus* venoms have also been performed [46,67,68]. More studies examining in vitro coagulotoxic venom effects have been performed on *B. taeniatus* [46,57,69,70], although the species’ venom remains relatively under-studied. 

In this study, we investigated the coagulotoxic venom action of 37 *Bothrops* venom samples (comprising 19 different species and numerous geographical localities) on human plasma and fibrinogen using an in vitro assay: thromboelastography. By using numerous geographical localities and testing many under-studied species, this study aims to provide a detailed description of the clotting action of these evolutionarily novel and clinically important venomous snakes. We hope our results can guide the future research and management of *Bothrops* bites, particularly from under-studied species (such as the arboreal and island-dwelling *Bothrops*) and/or snakes from under-studied regions.

## 2. Results

### 2.1. Venom Action on Human Plasma

**Figure 1:**
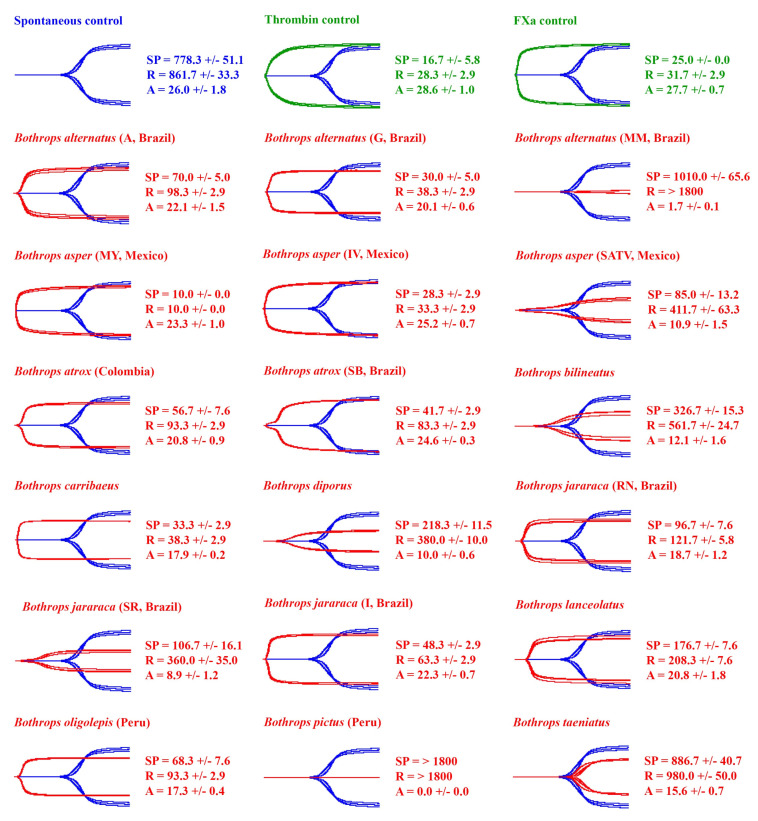
Thromboelastography traces showing the action of *Bothrops* venoms (red traces) on human plasma. A total of 18 representatives (red traces) of the 37 venoms examined (Table 1) are overlaid on top of the spontaneous control (blue traces) and ordered in the figure alphabetically. Three controls were performed: a negative control (spontaneous clotting time of plasma, blue traces), and two positive controls (thrombin control and FXa control, green traces). Three clotting parameters are shown: SP (split point—time until tracing splits, representing start of clotting), R (reaction time—time until amplitude = 2 mm, representing time until detectable clot), and A (Amplitude—width of tracing at latest time point, representing clot strength at latest time point). All values are mean ± standard deviation (*n* = 3). Each test lasted 1800 s, so > 1800 s indicates that the parameter was not recorded in this time. The spontaneous clotting control of human plasma (negative control) had an SP of 778.3 ± 51.1 s, R of 861.7 ± 33.3 s, and A of 26.0 ± 1.8 mm. Locality details for locality abbreviations used in this figure can be found in Table 1.

**Figure 2:**
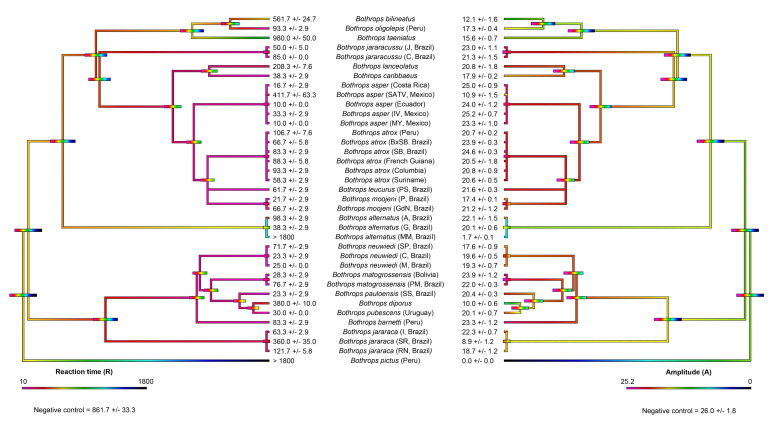
Ancestral state reconstructions of *Bothrops* venom clotting parameters from thromboelastography on human plasma. The parameters R (reaction time—time until amplitude = 2 mm, representing time until detectable clot in seconds) (**left**) and A (amplitude—width of tracing at latest time point, representing clot strength at latest time point in mm) (**right**) are shown. All values are mean ± standard deviation (*n* = 3) and spontaneous clotting control values are shown below each phylogeny. The colour gradient ranges from violet to black, with violet representing faster clotting times (**left** hand side) and stronger clots (**right** hand side). Note: due to the high dynamicity of venom evolution, the node bar ranges quickly become broad as one moves down the tree. The phylogeny was produced using timetree.org and updated with information from Alencar et al. [71], Carrasco et al. [72], and Fenwick et al. [73]. Each test lasted 1800 s, so R >1800 s indicates that R was not recorded in this time. Note: although *B. alternatus* (MM, Brazil) has an R > 1800 s, a weak clot was still observed (A = 1.7 ± 0.1) (R is only recorded if A > 2 mm). The spontaneous clotting control of human plasma (negative control) had a reaction time (R) of 861.7 ± 33.3 and amplitude (A) of 26.0 ± 1.8 mm. Locality details for locality abbreviations used in this figure can be found in Table 1.

The spontaneous clotting control of human plasma (negative control) had a reaction time (R) of 861.7 ± 33.3 and an amplitude (A) of 26.0 ± 1.8 mm (Figure 1 and Figure 2). The positive controls R was 28.3 ± 2.9 s (thrombin control) and 31.7 ± 2.9 s (FXa control), while A was 28.6 ± 1.0 mm (*n* = 3) (thrombin control) and 27.7 ± 0.7 mm (FXa control) (Figure 1).

Ancestral state reconstructions (Figure 2) highlight the clotting activity of *Bothrops* across the phylogeny. A diverse range of clotting activity on human plasma was seen in the *Bothrops* samples tested, with R ranging from 10 to 1800 s (machine maximum reading), and A ranging from 0 to 25.2 mm. Consistent with procoagulant toxicity being a defining feature of *Bothrops* venoms, most venoms quickly formed strong clots, such as those of the positive controls (Figure 1 and Figure 2). Although the clots were strong, no venom reached the clotting strengths of the controls. Despite most species forming quick and strong clots, there was variability observed between them (Figure 1 and Figure 2). In particular, significant variation in the relative procoagulant potency was observed between *B. alternatus*, *B. asper*, *B. atrox*, and *B. jararaca*, and the localities. Conspicuously slower, but still procoagulant, was *B. lanceolatus* (Figure 1 and Figure 2). 

Not all species venom formed fast, strong clots (Figure 1 and Figure 2). Consistent with *Bothrops* evolving from anticoagulant snakes, the most basal species, *B. pictus*, prevented plasma from clotting before the machine maximum time was reached, thereby displaying extremely potent anticoagulant toxicity. Intriguingly, while the other two studied localities of *B. alternatus* were potently procoagulant, the Mogi Mirim, Brazil locality was anticoagulant, thus representing a secondary loss of the derived procoagulant trait. The venom did clot plasma, albeit weakly (A = 1.7 mm), but only started clotting plasma after an extended period of time (SP = 1010 ±/ 65.6 s, shown on Figure 1); thus, it was deemed anticoagulant. Note that R was not recorded for the Mogi Mirim, Brazil locality (R > 1800) as A was less than 2 mm. While one arboreal species (*B. oligolepis*) acted in a procoagulant manner, rapidly producing a strong clot, the two others studied (*B. bilineatus* and *B. taeniatus*) displayed unique patterns in the plasma thromboelastography studies (Figure 1 and Figure 2). The arboreal species *B. taeniatus* marginally extended clotting time past that of the negative (spontaneous clotting) control, but conspicuously, clot strength was greatly reduced. Despite a clot being registered before the negative control (spontaneous clotting time) for another arboreal species, *B. bilineatus*, the clot strength was, like that of *B. taeniatus*, greatly reduced. Similarly, despite clotting being registered at time values shorter than those of the negative (spontaneous clotting) control, only weak clots were formed for the San Andres, Mexico locality of *B. asper*, *B. diporus*, and the São Roque locality of *B. jararaca* (Figure 1 and Figure 2).

### 2.2. Venom Action on Human Fibrinogen

**Figure 3:**
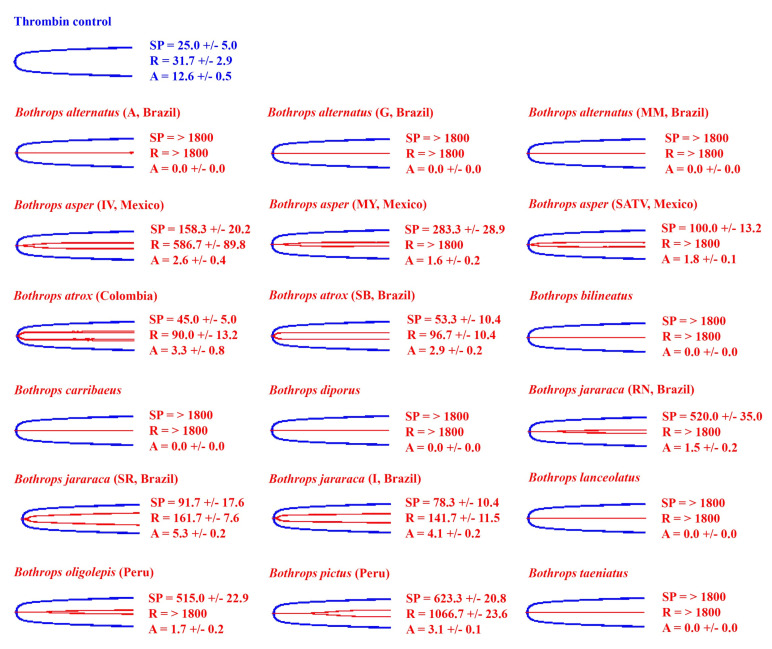
Thromboelastography traces showing the action of *Bothrops* venoms (red traces) on human fibrinogen. A total of 18 representatives (red traces) of the 37 venoms examined (Table 1) are overlaid on top of the thrombin control (blue traces) and ordered in the figure alphabetically. Three clotting parameters are shown: SP (split point—time until tracing splits, representing start of clotting), R (reaction time—time until amplitude = 2 mm, representing time until detectable clot), and A (amplitude—width of tracing at latest time point, representing clot strength at latest time point). All values are mean ± standard deviation (*n* = 3). Each test lasted 1800 s, so >1800 s indicates that the parameter was not recorded in this time. The thrombin control had an SP of 25.0 ± 5.0 s, R of 31.7 ± 2.9 s, and A of 12.6 ± 0.5 mm. Locality details for locality abbreviations used in this figure can be found in Table 1.

**Figure 4:**
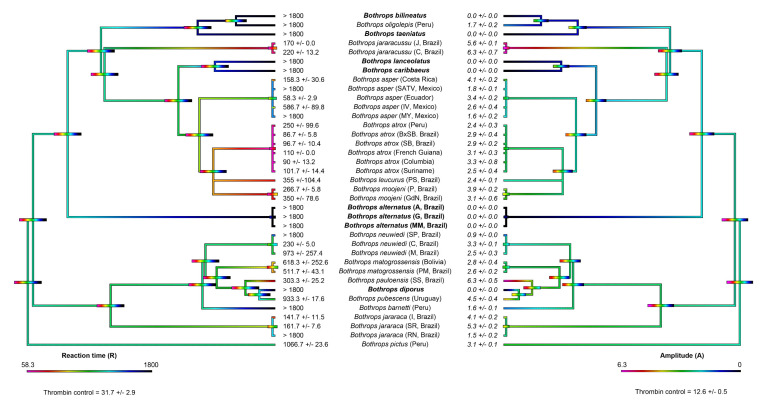
Ancestral state reconstructions of *Bothrops* venom clotting parameters from thromboelastography on human fibrinogen. The parameters R (reaction time—time until amplitude = 2 mm, representing time until detectable clot in seconds; **left**) and A (amplitude—width of tracing at latest time point, representing clot strength at latest time point in mm; **right**) are shown. All values are mean +/− standard deviation (*n* = 3) and thrombin control values are shown below each phylogeny. The colour gradient ranges from violet to black, with violet representing faster clotting times (**left** hand side) and stronger clots (**right** hand side). Note: due to the high dynamicity of venom evolution, the node bar ranges quickly become broad as one moves down the tree. Each test lasted 1800 s, so R > 1800 s indicates that R was not recorded in this time. Bold species names indicate no R parameter was recorded in the test time; thus, R = >1800, and the A parameter = 0 mm (no clot observed, TEG trace flatlined). Note: although *Bothrops barnetti* (Peru) and *B. oligolepis* (MM, Brazil) have an R > 1800 s, a weak clot was still observed (A = 1.6 ± 0.1 and 1.7 ± 0.2, respectively; R is only record if A > 2 mm). The phylogeny was produced using timetree.org and updated with information from Alencar et al. [71], Carrasco et al. [72], and Fenwick et al. [73]. The thrombin control had an R of 31.7 ± 2.9 s and A of 12.6 ± 0.5 mm. Locality details for locality abbreviations used in this figure can be found in Table 1.

In contrast to recalcified plasma, fibrinogen does not spontaneously clot. Thus, all the results were compared against the thrombin control (positive control). The thrombin control had a reaction time (R) of 31.7 ± 2.9 s and an amplitude (A) of 12.6 ± 0.5 mm (Figure 3 and Figure 4). 

In order to ascertain whether the species noted above that lack true procoagulant toxicity (San Andres, Mexico locality of *B. asper*, *B. diporus*, São Roque locality of *B. jararaca*, and the arboreal species *B. bilineatus* and *B. taeniatus*) clot fibrinogen, and to ascertain if this direct action on fibrinogen was a background activity for other species, thromboelastography studies were repeated using purified fibrinogen in place of plasma. Ancestral state reconstructions (Figure 4) were used to highlight the clotting activity of *Bothrops* across the phylogeny.

Consistent with the plasma patterns, the San Andres, Mexico locality of *B. asper* and the São Roque locality of *B. jararaca* directly clotted fibrinogen in a pseudo-procoagulant manner, forming weak fibrin clots relative to the thrombin control (Figure 3 and Figure 4). Therefore, these species lack the true procoagulant trait of factor activation (representing a secondary loss of this trait), and instead, only produce the pseudo-procoagulant form of clotting. 

Consistent with pseudo-procoagulant action on fibrinogen being an evolutionary relic for most species, there was no clear organismal phylogenetic pattern observed for fibrinogen clotting for the other species that displayed procoagulant venom on human plasma; venoms that possess pseudo-procoagulant action are apparently randomly distributed across the phylogeny, and many species lack this activity (Figure 3). Additionally, and also consistent with pseudo-procoagulant action on fibrinogen being an evolutionary relic, is that most venoms acting in this manner are slower than that of anticoagulant vipers, which use action upon fibrinogen as part of their predatory arsenal [9,55,74,75].

### 2.3. Fibrinogen Destruction Venom Activity 

**Figure 5:**
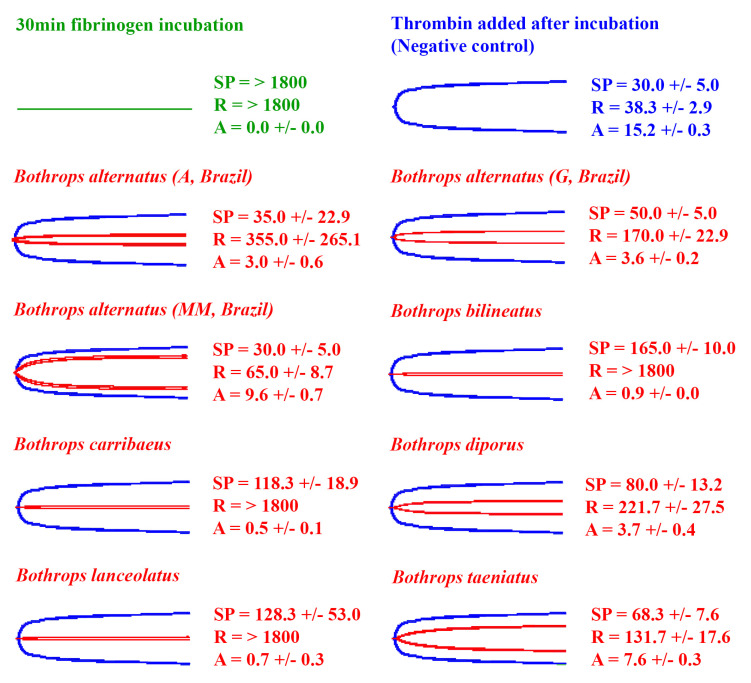
Thromboelastography traces showing the action of *Bothrops* venoms that did not clot fibrinogen (8/37 venoms) in a fibrinogen destruction assay. Venoms are ordered alphabetically in the figure. For each test, fibrinogen was incubated with venom for 30 min before the addition of thrombin in a Claussian protocol. The green trace represents the trace of each sample after 30 min incubation (no clot formed). The positive control (thrombin control) is shown in blue and venom samples (red traces) are overlaid on top of the thrombin control (blue traces). Three clotting parameters are shown: SP (split point—time until tracing splits, representing start of clotting), R (reaction time—time until amplitude = 2 mm, representing time until detectable clot), and A (amplitude—width of tracing at latest time point, representing clot strength at latest time point). All values are mean ± standard deviation (*n* = 3). Each test lasted 1800 s, so >1800 s indicates that the parameter was not recorded in this time. The thrombin control had an SP of 30.0 ± 5.0 s, R of 38.3 ± 2.9 s, and A of 15.2 ± 0.3 mm. Locality details for locality abbreviations used in this figure can be found in Table 1.

The eight venoms that did not induce a measurable clot on fibrinogen (A = 0, R = > 1800, flatline on TEG trace) (Figure 3 and Figure 4) were further tested for their ability to cleave fibrinogen in a non-clotting, destructive manner (Figure 5). This was undertaken using the Claussian method [76], whereby after the incubation of venom with fibrinogen, a vast excess of thrombin was added. Any increase in clotting time or decrease in clot strength observed is due to a depletion of fibrinogen levels.

All eight venoms that did not clot fibrinogen (*B. diporus*, *B. alternatus* (Araraquara, Guararena, and Mogi Mirim, Brazil), *B. lanceolatus*, *B. caribbaeus*, *B. bilineatus*, and *B. taeniatus*) exhibited fibrinogen destruction to varying degrees (Figure 5). The island dwellers (*B. lanceolatus* and *B. caribbaeus*) and the arboreal *B. bilineatus* were the most potent fibrinogen destroyers (A < 1.0 mm), while *B. alternatus* (Mogi Mirim, Brazil) was the weakest (A = 9.6 ± 0.7 mm). Like the pseudo-procoagulant fibrinogen clotting, no clear organismal phylogenetic pattern was observed for fibrinogen destruction, with venoms that possess this action apparently randomly distributed across the phylogeny (Figure 3). 

## 3. Discussion

By undertaking the most comprehensive study to date of *Bothrops* coagulotoxic effects (37 venoms spanning 19 species and covering the full taxonomical and ecological diversity, including arboreal and island species), this study produced data with tangible, real-world applications by providing information regarding potential clinical effects, while also contributing to the theoretical knowledge of their evolutionary biology. Procoagulant toxicity was shown to be a defining trait of the genus (Figure 1 and Figure 2). Although studies on specific coagulation factor activation were not performed, the strong procoagulant toxicity is likely achieved through coagulation factor activation (e.g., prothrombin and FX activation) [36,38,39,40,41,42,43]. Consistent with previous studies using more limited datasets [15,16,17,43] interspecific and intraspecific variations were noted. 

A conspicuous result was the documentation of *B. caribbaeus* and *B. lanceolatus* as being procoagulant in this study, when prior work had suggested that these species lacked this trait [66,77]. However, in both prior studies, recalcification was not undertaken for the citrated plasma, as they followed a 1983 protocol that does not include calcium and, consequently, does not reproduce physiological conditions [78]. This is a flaw in the study design, as it has been shown that some *Bothrops* venoms themselves are calcium-dependent in their action [42,43]. Similarly, in neither study was the phospholipid cofactor added, which has also been shown to be an important co-factor for many procoagulant venoms [5,79]. Thus, in the absence of calcium and phospholipid, little, if any, venom action would be evident; this was clearly the case in the prior *B. caribbaeus* and *B. lanceolatus* studies as both venoms were procoagulant upon recalcified plasma in this study, and produced strong, stable clots (Figure 1 and Figure 2). Furthermore, Bourke et al. [15] tested BothroFav (a monospecific *B. lanceolatus* antivenom) efficacy on procoagulant *B. atrox* and *B. asper* and showed that the antivenom was able to neutralise the procoagulant venom effects, specifically those of *B. atrox*. This suggests that procoagulant toxins are present in *B. lanceolatus*, as horses immunised with *B. lanceolatus* venom produce an antivenom (BothroFav) that can neutralise such toxins. Overall, the procoagulant activity of the island-dwelling *Bothrops* in this study is interesting and deserves further attention in future studies.

The most basal species, *B. pictus* [71,80], was like most non-*Bothrops* American pit vipers [37,55,56,81] in that it lacked procoagulant venom; it was anticoagulant on human plasma (Figure 1 and Figure 2). This result might be indicative of the common ancestor of all *Bothrops* having a distinct anticoagulant venom action compared to the often procoagulant action seen in the more derived *Bothrops*. Indeed, the limited studies that have been performed on the sister genus *Bothrocophias* indicate that their venom has low coagulant activity [82,83]. Interestingly, despite being anticoagulant on human plasma, when tested on isolated fibrinogen, *B. pictus* venom induced a weak clot (A = 3.1 ± 0.1 mm) after an extended period (R = 1066.7 ± 23.6) (Figure 3). This indicates that pseudo-procoagulant (aka ‘thrombin-like’) enzymes are present in the venom, which confirms previous studies [84]. When injected into prey items, the pseudo-procoagulant enzymes likely act synergistically with other anticoagulant toxins, consuming clotting factors to produce an anticoagulant pathology. 

Furthermore, underscoring the dynamic nature of venom diversification, the characteristic *Bothrops* procoagulant venom trait was secondarily lost in several species. This includes the derived arboreal species *B. bilineatus* and *B. taeniatus* (Figure 1 and Figure 2). This is consistent with previous reports [46,57,67,70]. Note that previous literature on *B. bilineatus* subspecies found that *B. b. bilineatus* clotted human plasma [46,67], while *B. b. smaragdinus* did not [67]. The subspecies of the present studies venom sample is unknown. Some terrestrial species have also secondarily lost the procoagulant venom trait: the Mogi Mirim, Brazil locality of *B. alternatus*; the San Andres, Mexico locality of *B. asper*; *B. diporus*; and the São Roque of *B. jararaca*. The divergence within *B. alternatus* is consistent with previous reports on other localities that found extreme variation between venoms in Brazil [70], which is also consistent with this wide-ranging species, in fact, being a species complex.

Unlike procoagulant toxicity, which is well defined as being a potent predatory weapon through stroke induction, it has been suggested that the pseudo-procoagulant direct action upon fibrinogen to produce weak, short-lived fibrin clots is an evolutionary relic and does not play a role in prey capture, as it is much slower in action than procoagulant factor activation [36,43]. However, as this theory was advanced through datasets containing limited venom diversity, the role has remained enigmatic in the absence of a comprehensive study such as this one. The results demonstrate that there was a lack of phylogenetic signal for this trait in that the variations were random between species and locality (Figure 3 and Figure 4). This, indeed, lends supports to the hypothesis that this trait is an ancestral relic and, assuming no ecological difference among the *Bothrops* species examined, not subjected to purifying selection pressure. However, as has been noted in clinical cases [24,52], human bite victims survive long enough for the additional depletion of fibrinogen to contribute to the consumptive coagulopathy; therefore, the documentation of the species with the greatest relative potency in this regard will contribute to the body of knowledge, which is useful for designing evidence-based clinical management strategies. The species with the most relatively potent action (small R and small A values) in this regard were the terrestrial species *B. jararacussu* (all tested), *B. asper* (Costa Rica and Ecuador only amongst those tested in this study), *B. atrox* (all tested), *B. leucurus*, *B. moojeni* (all tested)*, B. neuwiedi* (Curitiba, Brazil only amongst those tested in this study), *B. pauloensis*, and *B. jararaca* (all tested except Rio Negrino). This suggests that in clinical cases, these species may produce additional fibrinogen depletion on top of that consumed by the procoagulant mode of action.

In addition, some other species showed the ability to destructively (non-clotting) cleave fibrinogen, which would also contribute to fibrinogen depletion (Figure 5). *Bothrops diporus*, *B. alternatus* (Araraquara, Guararena, and Mogi Mirim, Brazil), *B. lanceolatus*, *B. caribbaeus*, *B. bilineatus*, and *B. taeniatus* all displayed the ability to destroy fibrinogen. The most potent were the island species *B. lanceolatus* and *B. caribbaeus* and the arboreal species *B. bilineatus*. Interestingly, despite destroying fibrinogen (anticoagulant effect) *B. lanceolatus* and *B. caribbaeus* were also potently procoagulant on human plasma (Figure 1, Figure 2 and Figure 5). This is counter-intuitive: procoagulant toxins work to produce a clot while fibrinogen-destroying toxins work to stop the production of a clot. The observed clot on human plasma is likely reflective of the procoagulant toxins taking over and producing fibrin before fibrinogen destruction can occur. Other contrasting venom actions were seen for the Guararena and Araraquara locality *B. alternatus*, which produced strong clots in human plasma, but also destroyed fibrinogen (Figure 1, Figure 2 and Figure 5). 

Interestingly, extensive variation was shown within *B. alternatus*, with the Mogi Mirim locality being much weaker than the other two localities in the fibrinogen destruction trait (Figure 5), despite lacking the procoagulant venom trait as shown in human plasma tests (Figure 1 and Figure 2). This suggests that toxins that inhibit upstream coagulation factors, rather than directly affect fibrinogen, are involved in producing the anticoagulant effects on human plasma observed for the *B. alternatus* Mogi Mirim locality venom. For the other species that lacked a procoagulant trait, the terrestrial species *B. diporus* and the arboreal species *B. bilineatus* and *B. taeniatus*, have upregulated the ability to directly cleave fibrinogen in a destructive (non-clotting) manner. This suggests that, at least in these venoms, the action may have a role in prey capture, with these snakes having reverted back to the ancestral fibrinogen-depleting anticoagulant condition. In addition, relative to procoagulant species, the arboreal species may have greater reliance upon the neurotoxic activity documented for those venoms [85,86,87] to subjugate prey. A lack of procoagulant activity might be beneficial in allowing neurotoxins to spread throughout the body, as localised clots might “trap” neurotoxins, preventing them from spreading. This theory of toxin synergism and clots “trapping” toxins is discussed in Jackson et al. [88]. Furthermore, *B. bilineatus* and *B. taeniatus* feed on a variety of vertebrates [14,89,90]; thus, rather than neurotoxicity evolving to target a particular prey type, neurotoxins may have evolved in the venom to incapacitate prey items quickly, decreasing the chance of prey dropping from the trees and escaping. This hypothesis, however, needs to be tested by ascertaining relative neurotoxic effects on prey-lineage targets.

The secondary change from procoagulant to anticoagulant cleavage of fibrinogen, whether in a destructive manner or a pseudo-procoagulant manner, is almost certainly due to differential selection pressure for the species that display these traits. The arboreal species are under clear divergent selection pressure for prey-escape potential, thus providing a hypothesis regarding the change in venom phenotype to the destructive fibrinogenolytic form of anticoagulation. However, there is an enigmatic question of what selection pressures (e.g., changes in prey preference or changes in prey-escape potential) led to the divergence—relative to other terrestrial *Bothrops*—for *B. diporus* to convergently evolve with these arboreal species the destructive fibrinogenolytic form of anticoagulation. What is also enigmatic is the question of why the San Andres, Mexico locality of *B. asper* and the São Roque locality of *B. jararaca* convergently evolved relative to each other to lose the procoagulant trait, and instead, have the pseudo-procoagulant fibrinogenolytic venom phenotype. These results, therefore, provide a starting point for natural history observations to reconstruct the selection pressures leading from these species, which are nested deep within the procoagulant *Bothrops* clade, to diverge to a fibrinogenolytic venom phenotype.

This documentation of extreme coagulotoxicity diversity across the *Bothrops* genus is a tangible benefit, as clinical observation of envenomated patients corroborate these results, with patients experiencing the haemostatic disorders of unclottable blood and systemic bleeding [23,24,25,91,92]. 

Understanding the caveats of a study are important so that appropriate conclusions can be made. An important caveat in this study is that all tests are in vitro, without flow of blood and without multiple systems interacting; hence, the conclusion of the results needs to be interpreted with this in mind. In vitro studies are effective for investigating specific venom actions upon the coagulation cascade, such as in the present study. As in vivo systems are complex and dynamic, in vitro results may not line up with in vivo results. An important note, however, is that this study was undertaken using human plasma and; thus, while in vitro, it may produce results more suggestive of potential human envenomation effects than in vivo animal-model studies, if the venoms have a differential effect between humans and animal-models. This is, indeed, recognised as a critical issue and a limitation of the applicability of animal-based research for human medicine [93]. Regardless, future in vivo work should be conducted before making any therapeutic recommendations regarding clinical care. Lastly, this study only tested venom on human plasma and fibrinogen, yet some *Bothrops* venoms have been shown to have different action when tested on the plasma of different prey types [43]. Although it is beyond the scope of this study, future studies should test a wide range of *Bothrops* venom on non-human animal plasmas to test for differences in venom action among prey types, in order to provide information about evolutionary selection pressures leading to changes in the venom phenotype.

Overall, this study vastly improves our fundamental knowledge base on the coagulotoxic action of *Bothrops* venoms. We hope this wealth of new knowledge for toxinologists will fuel many future studies, as well as provide a solid platform for evolutionary biologists to use alongside natural history observations of these fascinating snakes.

## 4. Materials and Methods

### 4.1. Venom Sample Preparation

Venom work was undertaken under University of Queensland Animal Ethics Approval 2021/AE000075. All venoms used in the study were in lyophilised form and obtained from the University of Queensland’s Venom Evolution Lab’s long-term venom collection, venom suppliers, and collaborators. Thirty-seven venoms were used in the study, including nineteen different species and numerous geographical variants (Table 1). 

Venoms were prepared to a 1 mg/mL stock solution (50:50 double deionised water (ddH20):glycerol) using a Thermo Fisher Scientific™ NanoDrop 2000 (Waltham, MA, USA) at 280 nm wavelength, and stored at −80 °C until experimentation, during which they were stored at −20 °C. This storage limited enzyme degradation, as did the use of glycerol in venom stocks, which function to stabilise enzymes.

### 4.2. Plasma and Fibrinogen Preparation 

Human plasma work was performed under University of Queensland Biosafety Approval #IBC134BSBS2015 and Human Ethics Approval #2016000256. The Australian Red Cross (44 Musk Street, Kelvin Grove, QLD 4059, Australia) supplied human platelet-poor plasma (3.2% citrated, O positive, Label # A540021261153) under research approval #16-04QLD-10. Once obtained, plasma was stored at −80 °C until aliquoted. Plasma was aliquoted into 1.5 mL tubes by defrosting in a water bath at 37 °C and aliquoting into 1.5 mL tubes in a biosafety cabinet, to avoid contaminating the plasma. Plasma was then stored at −80 °C until experimentation.

Human fibrinogen (Sigma Aldrich, St. Louis, MO, USA) was prepared to 4 mg/mL by diluting 100 mg of fibrinogen with Owren Koller (OK) buffer to a volume of 25 mL. This solution was then vortexed until solubilised; it was aliquoted, then the tubes were flash-frozen with liquid nitrogen and immediately stored at −80 °C. 

### 4.3. Thromboelastography Experiments

Two TEG^®^ 5000 Thrombelastograph^®^ Haemostasis Analyser systems were used concurrently, containing two reaction stations each. Natural cups and pins were placed into each channel and the heating plate set to 37 °C. All reagents were then pipetted into the cup as per our previously validated protocol [43]: 72 μL CaCl (25 mM stock solution Stago Cat# 00367); 72 μL phospholipid (Stago Cat# 00597), solubilised in Owren Koller (OK) buffer (Stago Cat #00360); 20 μL OK buffer; and 7 μL venom sample or control sample (negative control: 7 μL 50:50 ddH20: glycerol, thrombin control: 7 μL thrombin, or FXa control: 7 μL FXa). A total of 189 μL plasma or fibrinogen, which had been thawed in a water bath at 37 °C for 5 min, was taken out of the water bath and then pipetted into each cup, after which the 360 μL sample was pipette-mixed and the machine was run for 30 min. For each cup, the time from pipetting the plasma into the cup until the start of each test (lifting the cup into the test machine and pressing start) was 10 s. Each venom test was performed in triplicate (*n* = 3).

Venoms that did not clot fibrinogen (the TEG trace was a flatline) were immediately tested for fibrinogen destruction via a Claussian method [76], using our previously validated protocol [94]. Fibrinogen destruction tests were conducted because fibrinogen does not spontaneously clot, like human plasma; thus, a flatline could indicate that the venom either has no action on fibrinogen or that the venom destroys fibrinogen. In this test, 7 μL of thrombin was added to the cups, which already had a 360 μL sample in them from the prior test. Then, the whole solution was pipette mixed and run again for 30 min. The results from the venom runs were compared to the negative control, where a blank (7 μL ddH20/glycerol) replaced the venom, and thrombin induced a clot upon fibrinogen in the absence of venom. Note that some venoms that produced a flatline on the initial fibrinogen destruction test qualitatively produced micro ‘clots’ (small strands). These were noted for four venoms (*B. alternatus* (Mogi Mirim, Brazil), *B. alternatus* (Guararena, Brazil), *B. alternatus* (Araraquara, Brazil), and *B. lanceolatus* (Martinique)) and were ignored in the study; this is because they were not recorded on the machine and, therefore, were unlikely to be clinically significant. 

The parameters produced by thromboelastography include split point (SP), reaction time (R), amplitude (A) and maximum amplitude (MA). SP is the time until the tracing splits, representing the first formation of fibrin strands. R is the time until the first detectable clot appears (defined by the machine as enough resistance to produce an amplitude >2 mm). R is the most often-used variable to indicate clot initiation. R is where classic coagulation assays, such as prothrombin time (PT) and partial thromboplastin time (PTT) assays are completed; thus, TEG provides a more detailed picture of coagulation than these assays. Both A and MA represent clot strength. Clot strength is measured as the width of the tracing in mm (the greater the width, the greater the clot strength). A is the strength of the clot at the latest time point, while MA is the maximum strength of the clot reached during the run. MA is only recorded after A > 2 mm (A is equal to MA until MA is determined). MA is calculated using the small deviation method (time = 3 min), in which MA is only calculated if itdoes not deviate more than 1 mm for at least 3 min, thus explaining why A is sometimes >MA. 

For a detailed description of how TEG works, see Supplementary File S1. 

### 4.4. Data Analysis

Phylogenetic trees were initially produced using TimeTree (TimeTree.org) and exported to Mesquite (version 3.61), where additional branches for localities and certain species were made manually. TimeTree collates data from published studies to produce a phylogeny from the specified genus which, in this study, is *Bothrops*. The resulting tree was missing two of the species analysed in the study: *Bothrops barnetti* and *B. oligolepis*. The missing species were manually added to the tree and their branch lengths estimated, based on phylogenies from [71,72,73]. The Mesquite tree file (.phy file) is available in the Supplementary Materials (Supplementary File S2). The tree was then imported into the statistical software R (version 3.6.1) using the APE package [95]. The contMAP function of the phytools package [96] was used to estimate ancestral states, using maximum likelihood, and to visually represent the presented trait over the tree. Four trees were produced: a tree produced from human plasma reaction time data, a tree produced from human plasma amplitude data, a tree produced from fibrinogen reaction time data, and a tree produced from fibrinogen amplitude data. Each data file used to produce the trees can be found in the Supplementary Materials (Supplementary File S3). The R code is also available in the Supplementary Materials (Supplementary File S4). The trees were then exported from R and edited with Adobe Photoshop (version 21.2.11) and Adobe Acrobat Pro DC (version 2021.011.20039) to produce Figure 2 and Figure 4. Editing consisted of positioning the reaction time and amplitude trees opposite each other, adding details of species names, and adding the mean ± standard deviation of R and A values for each respective branch.

Thromboelastography traces were exported from the TEG 5000, and Figure 1, Figure 3 and Figure 5 produced in Adobe Photoshop. All thromboelastography raw data for each venom can be found in the Supplementary Materials (Supplementary File S5), with the following parameters supplied: SP, R, Angle, A, and MA. Note that in the raw data, A is never exactly 0 mm as the sample always induces some resistance. In this study we classed all A values as 0 mm if the trace was a flatline. Additionally, note that for consistency, Amplitude (A) is used in Figure 1, Figure 2, Figure 3, Figure 4 and Figure 5 instead of Maximum Amplitude (MA), since for some venoms, MA could not be calculated (MA is only calculated if A > 2.0 mm). Furthermore, R was used instead of SP in phylogenetic trees (Figure 2 and Figure 4) as R represents the time until detectable clot formation rather than just the first deposition of fibrin strands (SP). R was deemed a more meaningful parameter to plot. 

## Figures and Tables

**Table 1:** *Bothrops* venoms used in the study with additional details supplied: whether venoms are from single individuals or pooled from multiple individuals (*n* values are supplied if known), locality, sex, age, and source (captive-born or wild-caught snakes). Note: captive-born snakes originate from snakes from the specified locality. Locality abbreviations are the abbreviation for each locality used in the study (“–” = no abbreviation given).

Species	Pooled (*n*) or Individual	Locality	Locality Abbreviation in Figures (If Used)	Sex	Approx Age (yrs)	Source, and Wild-Caught or Captive Bred Stock
*B. alternatus*	Individual	Mogi Mirim–SP, Brazil	MM, Brazil	F	9	Instituto Butantan, wild-caught in 2009
*B. alternatus*	Individual	Guararena–SP, Brazil	G, Brazil	F	7	Instituto Butantan, captive-born in 2011, 17a specimen from litter
*B. alternatus*	Individual	Araraquara–SP, Brazil	A, Brazil	F	5	Instituto Butantan, wild-caught in 2013
*B. atrox*	Individual	São Bento–MA, Brazil	SB, Brazil	F	12	Instituto Butantan, wild-caught in 2009
*B. atrox*	Pooled (2)	Balbira–AM, Brazil x São Bento–MA, Brazil	BxSB, Brazil	F	8	Institute Butantan, captive-born in 2010, l1a specimen from litter
		Balbira–AM, Brazil x São Bento–MA, Brazil		F	8	Institute Butantan, captive-born in 2010, 7a specimen from litter
*B. atrox*	Pooled (66)	French Guiana	-	M + F	Adults	Latoxan, captive-born and wild-caught snakes
*B. atrox*	Pooled (*n* values not supplied)	Alto Marañon, Peru (Amazon rainforest)	Peru	UNKN	Adults	EFS, wild-caught
*B. atrox*	UNKN	Suriname	-	UNKN	Adults	Kentucky reptile zoo, unknown
*B. atrox*	UNKN	Colombia	-	UNKN	Adults	Kentucky reptile zoo, unknown
*B. asper*	Pooled (40)	Costa Rica (Pacific region)	Costa Rica	UNKN	Adults	JMG, wild-caught
*B. asper*	Pooled (2)	Ecuador	-	M	Adults	Latoxan, captive-born
*B. asper*	Individual	Mérida, Yucatán, Mexico	MY, Mexico	UNKN	Adult	UNAM, wild-caught
*B. asper*	Individual	San Andres, Tuxtla, Veracruz, Mexico	SATV, Mexico	UNKN	Adult	UNAM, wild-caught
*B. asper*	Individual	Ixtaczoquitlan, Veracruz, Mexico	IV, Mexico	UNKN	Young adult	UNAM, wild-caught
*B. barnetti*	Pooled (*n* values not supplied)	Talara, Department of Tubmes, Peru	Peru	UNKN	Adults	UNMSM, wild-caught
*B. bilineatus*	Pooled (2)	UNKN	-	F + F	Adults	M-toxins, imported
*B. caribbaeus*	UNKN	St. Lucia, West Indies	-	UNKN	Adults	Kentucky reptile zoo, wild-caught
*B. diporus*	UNKN	UNKN	-	UNKN	Adults	VEL, imported
*B. jararacussu*	Pooled (2)	Juquitiba–SP	J, Brazil	F	3	Instituto Butantan, wild-caught in 2015
		Juquitiba–SP		F	3	Instituto Butantan, wild-caught in 2015
*B. jararacussu*	Individual	Cubatão–SP	C, Brazil	F	3	Instituto Butantan, wild-caught in 2015
*B. jararaca*	Individual	Rio Negrino–SC	RN, Brazil	F	1	Instituto Butantan, wild-caught in 2017
*B. jararaca*	Individual	São Roque–SP	SR, Brazil	F	3	Instituto Butantan, wild-caught in 2015
*B. jararaca*	Individual	Ibiúna–SP	I, Brazil	F	3	Instituto Butantan, wild-caught in 2015
*B. lanceolatus*	UNKN	Martinique	-	UNKN	Adults	Latoxan, imported
*B. leucurus*	Pooled (3)	Porto Seguro–BA	PS, Brazil	F	3	Instituto Butantan, captive-born in 2016, 5a specimen from litter
		Porto Seguro–BA		F	3	Instituto Butantan, captive-born in 2016, 13a specimen from litter
		Porto Seguro–BA		M	3	Instituto Butantan, captive-born in 2016, 14a specimen from litter
*B. mattogrossensis*	Pooled (3)	Porto Murtinho–MS	PM, Brazil	F	6	Instituto Butantan, captive-born in 2012, 4a specimen from litter
		Porto Murtinho–MS		F	9	Instituto Butantan, wild-caught in 2009
		Porto Murtinho–MS		F	9	Instituto Butantan, wild-caught in 2009
*B. mattogrossensis*	UNKN	Bolivia	-	UNKN	Adults	VEL, imported
*B. moojeni*	Individual	Palmas–TO	P, Brazil	F	13	Instituto Butantan, wild-caught in 2005
*B. moojeni*	Pooled (2)	Gaúcha do Norte–MT	GdN, Brazil	F	8	Instituto Butantan, captive-born in 2010, 13a specimen from litter
		Gaúcha do Norte–MT		F	8	Instituto Butantan, captive-born in 2010, 5a specimen from litter
*B. neuwiedi*	Individual	Salto Pirapora–SP	SP, Brazil	M	6	Instituto Butantan, captive-born in 2013, 3a specimen from litter
*B. neuwiedi*	Individual	Curitiba–PR	C, Brazil	F	2	Instituto Butantan, wild-caught in 2017
*B. neuwiedi*	Individual	Munhoz–MG	M, Brazil	F	4	Instituto Butantan, wild-caught in 2015
*B. oligolepis*	Pooled (3)	La Merced, Chanchamayo, Peru (central rainforest región)	Peru	UNKN	Adults	UNMSM, wild-caught
*B. pauloensis*	Pooled (3)	São Simão–SP	SS, Brazil	F	6	Instituto Butantan, captive-born in 2012, 6a specimen from litter
		São Simão–SP		F	7	Instituto Butantan, captive-born in 2011, 2a specimen from litter
		São Simão–SP		F	7	Instituto Butantan, captive-born in 2011, 6a specimen from litter
*B. pictus*	Pooled (7)	Districts of Carabayllo and Comas, Peru	Peru	UNKN	Adults	UNMSM, wild-caught
*B. pubescens*	UNKN	Uruguay	-	UNKN	Adults	VEL, imported
*B. taeniatus*	UNKN	UNKN	-	UNKN	Adults	VEL, imported

EFS = Laboratory of Biochemistry of Proteins from Animal Venoms, Research and Development Center, Ezequiel Dias Foundation, Belo Horizonte, MG 30510-010, Brazil; JMG = José María Gutiérrez, Instituto Clodomiro Picado, Facultad de Microbiología, Universidad de Costa Rica, San José 11501, Costa Rica; UNAM = Alejandro Alagon, Departamento de Medicina Molecular y Bioprocesos, Instituto de Biotecnologa, Universidad Nacional Autónoma de México, Av. Universidad 2001, Cuernavaca, Morelos 62210, Mexico; UNNSM = Armando Yarleque, Universidad Nacional Mayor de San Marcos, Lima, Peru; VEL = Venom Evolution Lab, UQ, Australia.

## Data Availability

Raw data are available in Supplementary File S5.

## Supplementary Materials

The following supporting information can be downloaded at: https://www.mdpi.com/article/10.3390/toxins14050297/s1, Supplementary File S1: Thromboelastography approach; Supplementary File S2: .phy tree file; Supplementary File S3: Data files for trees; Supplementary File S4: R code used in analyses; Supplementary File S5: Thromboelastography raw data.
