# Supplementary material for: Clinical and Evolutionary Implications of Dynamic Coagulotoxicity Divergences in Bothrops (Lancehead Pit Viper) Venoms
Source: Toxins (Basel). 2022 Apr 22;14(5):297. doi: 10.3390/toxins14050297 (PMC9148167; doi:10.3390/toxins14050297)
Supplement: Supplementary file 1 [file toxins-14-00297-s001.zip › Supplementary file S1 (TEG theory).pdf]

Supplementary file S1:

Thrombelastography theory

Two TEG® 5000 Thrombelastograph® Hemostasis Analyzer Systems (TEG) were used to quantify the action of venom on blood clotting (both human plasma and fibrinogen clotting). This machine uses disposable cups and pins. The cup is filled with a 360 µl sample, containing plasma and reagents, and a pin immersed into the cup. The machine measures coagulation parameters by oscillating the cup (moving left and right) through an arc of 4°45´, with each rotation cycle lasting 10 seconds (Figure 1A). As a clot starts to form the cup and pin start to become bonded and torque is applied to the pin, causing the pin (and torsion wire within the pin) to move. The pins rotation is transmitted to the torsion wire and converted to an electrical signal by a mechanical-electrical transducer, which is sent to a computer and observed as a trace [1,2] (Figure 1B). With increasing coagulation, the bond becomes greater, increasing the torque on the pin (clot strength is directly related to pin movement).

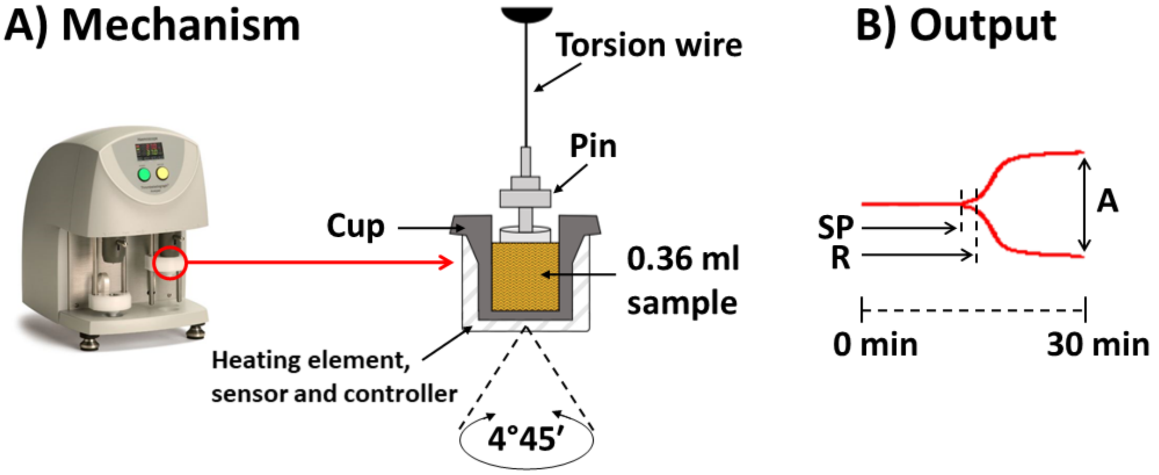

Figure 1. A) Image of a TEG 5000 machine used in this study (left) and an enlarged schematic of the TEG mechanism that measures clotting (right). The schematic was adapted from the TEG 5000 user manual. Assay details are described in the methods (see publication). B) An example of an output trace after completion of a 30 min run. The trace is labelled with time and clotting parameters SP (Split point – time until tracing splits, representing start of clotting), R (Reaction time – time until amplitude = 2mm, represent time until detectable clot), and A (Amplitude – width of tracing at latest time point, representing clot strength at latest time point). Note, the MA (Maximum amplitude – width of tracing at maximum width, represent maximum clot strength) is not shown. In this instance, however, A is = to MA as no lysis occurred, and thus MA is at the latest time point of the tracing.

The parameters produced by thrombelastography include split point (SP), reaction time (R), amplitude (A) and maximum amplitude (MA) (Figure 1B.). SP is the time until the tracing splits, representing the first formation of fibrin strands. R is the time until the first detectable clot (defined by the machine as enough resistance to produce an amplitude >2mm). R is the most often used variable to indicate clot initiation. R is where classic coagulation assays, such as prothrombin time (PT) and partial thromboplastin time (PTT) assays are completed, thus TEG provides a more detailed picture of coagulation then these assays. Both A and MA represent clot strength. Clot strength is measured as the width of the tracing in mm (the greater the width the greater the clot strength). A is the strength of the clot at the latest time point, while MA is the maximum strength of the clot reached during the run. MA is only recorded after A > 2mm (A is equal to MA until MA is determined). MA is calculated via the small deviation's method (time = 3min), in which MA is only calculated if the calculated does not deviate more than 1mm for at least 3 min, thus explaining why A is sometimes > MA.

References:

1. Whiting, D.; DiNardo, J.A. TEG and ROTEM: Technology and clinical applications. *American Journal of Hematology* **2014**, *89*, 228-232, doi:https://doi.org/10.1002/ajh.23599.
2. Ganter, M.T.; Hofer, C.K. Coagulation monitoring: Current techniques and clinical use of viscoelastic point-of-care coagulation devices. *Anesth. Analg.* **2008**, *106*, doi:https://10.1213/ane.0b013e318168b367.
