# Supplementary material for: Clinical and Evolutionary Implications of Dynamic Coagulotoxicity Divergences in Bothrops (Lancehead Pit Viper) Venoms
Source: Toxins (Basel). 2022 Apr 22;14(5):297. doi: 10.3390/toxins14050297 (PMC9148167; doi:10.3390/toxins14050297)
Supplement: Supplementary file 1 [file toxins-14-00297-s001.zip › Supplementary file S4 (R code).pdf]

Supplementary file S4: R code

To produce figures 1 and 3 the following was used: timetree.org, Mesquite software, R software, Microsoft excel, Adobe photoshop, and Adobe Acrobat Pro DC (See Data Analysis section in the paper for more details). R software was used to estimate ancestral states and visually represent the presented trait (thrombelastography parameters reaction time or amplitude) over the tree. Four R scripts were used, and the scripts are shown on the following pages.

- **Plasma Reaction Time Script** (pages 2-3) is the script used to estimate ancestral states and map the reaction time data from human plasma thrombelastography tests over the phylogenetic trees.

- **Plasma Amplitude Script** (pages 4-5) is the script used to estimate ancestral states and map the amplitude data from human plasma thrombelastography tests over the phylogenetic tree.

- **Fibrinogen Reaction Time Script** (pages 6-7) is the script used to estimate ancestral states and map the reaction time data from human fibrinogen thrombelastography tests over the phylogenetic tree.

- **Fibrinogen Amplitude Script** (pages 8-9) is the script used to estimate ancestral states and map the amplitude data from human fibrinogen thrombelastography tests over the phylogenetic tree.

Note, all scripts are similar except they differ in:

1) The data that is used

All scripts have the following five lines of code:

```
data<-read.csv(file.choose())

dat<-data

mapvar<-dat$var

names(mapvar)<-dat$species

tree<-read.tree(file.choose())
```

- Green highlight = code in which the data file is read into the R software. The data file (excel file .csv) contains species names and their mean Reaction Time or Amplitude value for fibrinogen or human plasma (depending on the script). For example, for the Plasma Reaction Time Script the .csv file would contain the species names and their mean Reaction Time values for human plasma. For data files see supplementary file 3: Data files for trees.

- Yellow highlight = code that reads the phylogenetic tree into the R software. The .phy file that is read into the software is the same for each of the four scripts (See supplementary file 2: .phy tree file)

2) Specific numbers in the script

All scripts have the following two lines of code

```
plot(setMap(asr,col=c(6,2,7,3,5,4,1)),lwd=3)
senci.contMap(setMap(asr,col=c(6,2,7,3,5,4,1)),lwd=5,min=10,max=1800)
```

Blue highlight = code that changes between Reaction Time and Amplitude Scripts.

For Reaction Time scripts "col=c(6,2,7,3,5,4,1)", while for Amplitude scripts "col=c(1,4,5,3,7,2,6)". This makes the colour gradient range from violet to black, except for Reaction Time scripts violet represents smaller numbers (small R values – fast clotting times) and for the Amplitude scripts violet represents larger numbers (large A values – strong clots).

Pink highlight = code that changes between each script. min = the minimum value in the dataset and max = the maximum value in the dataset. For example, the above 2 lines of code is taken from the Plasma Reaction Time Script. In this case the range of reaction time values in the dataset is 10 to 1800 seconds.

Plasma Reaction Time Script

senci.contMap is a slight modification of errorbar.contMap that trims 95% CIs of ancestral state reconstructions to a sensible range, e.g. for traits bound between 0 and 1
Example of code for implementing it would be as follows (lines separated by semicolons): pasr<-contMap(tree,mapdat,plot=F,lims=c(0,1)) ; plot(setMap(pasr,invert=T)) ; senci.contMap(setMap(pasr,invert=T),mini=0,maxi=1)

```
senci.contMap<-function(obj,...){
  if (hasArg(x))
    x <- list(...)$x
  else x <- setNames(sapply(1:Ntip(obj$tree), function(x, obj) {
    ii <- which(obj$tree$edge[, 2] == x)
    ss <- names(obj$tree$maps[[ii]][length(obj$tree$maps[[ii]])])
    obj$lims[1] + as.numeric(ss)/(length(obj$cols) - 1) *
      diff(obj$lims)
  }, obj = obj), obj$tree$tip.label)
  if (hasArg(scale.by.ci))
    scale.by.ci <- list(...)$scale.by.ci
  else scale.by.ci <- TRUE
  if (hasArg(lwd))
    lwd <- list(...)$lwd
  else lwd <- 14
  tree <- obj$tree
  aa <- fastAnc(tree, x, CI = TRUE)
  if (hasArg(min))#added lines here
    for (i in 1:length(aa$CI95[,1])){ #added lines here
      aa$CI95[i,1]<-ifelse(aa$CI95[i,1]<list(...)$min,list(...)$min,aa$CI95[i,1]) #added lines here
    } #added lines here
  else aa$CI95[,1]<-aa$CI95[,1] #added lines here
  if (hasArg(max)) #added lines here
    for (i in 1:length(aa$CI95[,2])){ #added lines here
      aa$CI95[i,2]<-ifelse(aa$CI95[i,2]>list(...)$max,list(...)$max,aa$CI95[i,2]) # added lines here
    } #added lines here
  else aa$CI95[,2]<-aa$CI95[,2] #added lines here
  xlim <- range(aa$CI95)
  if (xlim[2] > obj$lims[2] || xlim[1] < obj$lims[1]) {
    cat(paste("  -----\n  The range of the contMap object, presently (",
              round(obj$lims[1], 4), ",", round(obj$lims[2], 4),
              "), should be equal to\n  or greater than the range of the CIs on ancestral states: (",
              round(xlim[1], 4), ",", round(xlim[2], 4), ").\n",
              sep = ""))
    cat(paste("  To ensure that your error bars are correctly plotted, please recompute your\n",
              "  contMap object and increase lims.\n  -----\n",
              sep = ""))
  }
  d <- diff(obj$lims)
  if (scale.by.ci) {
    v <- aa$CI95[, 2] - aa$CI95[, 1]
    v <- v/max(v)
  }
  else v <- rep(0.5, tree$Nnode)
```

```
  n <- length(obj$cols) - 1
  lastPP <- get("last_plot.phylo", envir = .PlotPhyloEnv)
  h <- max(nodeHeights(tree))
  for (i in 1:tree$Nnode) {
    ii <- round((aa$CI95[i, 1] - obj$lims[1])/d * n)
    jj <- round((aa$CI95[i, 2] - obj$lims[1])/d * (n + 1))
    cols <- obj$cols[ii:jj]
    add.color.bar(leg = 0.1 * h * v[i], cols = cols, prompt = FALSE,
                  x = lastPP$xx[i + Ntip(tree)] - 0.05 * h * v[i],
                  y = lastPP$yy[i + Ntip(tree)], title = "", subtitle = "",
                  lims = NULL, lwd = lwd)
  }
}
library(ape)
library(maps)
library(phytools)
library(tree)

data<-read.csv(file.choose())
dat<-data
mapvar<-dat$var
names(mapvar)<-dat$species
tree<-read.tree(file.choose())
asr<-contMap(tree,mapvar,plot=F)
plot(setMap(asr,col=c(6,2,7,3,5,4,1)),lwd=3) # Reverse order of colour numbers as want blue to be high SP and red low SP
senci.contMap(setMap(asr,col=c(6,2,7,3,5,4,1)),lwd=5,min=10,max=1800)
```

Plasma Amplitude Script

senci.contMap is a slight modification of errorbar.contMap that trims 95% CIs of ancestral state reconstructions to a sensible range, e.g. for traits bound between 0 and 1
Example of code for implementing it would be as follows (lines separated by semicolons): pasr<-contMap(tree,mapdat,plot=F,lims=c(0,1)) ; plot(setMap(pasr,invert=T)) ; senci.contMap(setMap(pasr,invert=T),mini=0,maxi=1)

```
senci.contMap<-function(obj,...){
 if (hasArg(x))
   x <- list(...)$x
 else x <- setNames(sapply(1:Ntip(obj$tree), function(x, obj) {
   ii <- which(obj$tree$edge[, 2] == x)
   ss <- names(obj$tree$maps[[ii]][length(obj$tree$maps[[ii]])])
   obj$lims[1] + as.numeric(ss)/(length(obj$cols) - 1) *
     diff(obj$lims)
 }, obj = obj), obj$tree$tip.label)
 if (hasArg(scale.by.ci))
   scale.by.ci <- list(...)$scale.by.ci
 else scale.by.ci <- TRUE
 if (hasArg(lwd))
   lwd <- list(...)$lwd
 else lwd <- 14
 tree <- obj$tree
 aa <- fastAnc(tree, x, CI = TRUE)
 if (hasArg(min))#added lines here
   for (i in 1:length(aa$CI95[,1])){ #added lines here
     aa$CI95[i,1]<-ifelse(aa$CI95[i,1]<list(...)$min,list(...)$min,aa$CI95[i,1]) #added lines here
   } #added lines here
 else aa$CI95[,1]<-aa$CI95[,1] #added lines here
 if (hasArg(max)) #added lines here
   for (i in 1:length(aa$CI95[,2])){ #added lines here
     aa$CI95[i,2]<-ifelse(aa$CI95[i,2]>list(...)$max,list(...)$max,aa$CI95[i,2]) # added lines here
   } #added lines here
 else aa$CI95[,2]<-aa$CI95[,2] #added lines here
 xlim <- range(aa$CI95)
 if (xlim[2] > obj$lims[2] || xlim[1] < obj$lims[1]) {
   cat(paste("  -----\n  The range of the contMap object, presently (",
         round(obj$lims[1], 4), ",", round(obj$lims[2], 4),
         "), should be equal to\n  or greater than the range of the CIs on ancestral states: (",
         round(xlim[1], 4), ",", round(xlim[2], 4), ").\n",
         sep = ""))
   cat(paste("  To ensure that your error bars are correctly plotted, please recompute your\n",
         "  contMap object and increase lims.\n  -----\n",
         sep = ""))
 }
 d <- diff(obj$lims)
 if (scale.by.ci) {
   v <- aa$CI95[, 2] - aa$CI95[, 1]
   v <- v/max(v)
 }
```

```
  else v <- rep(0.5, tree$Nnode)
  n <- length(obj$cols) - 1
  lastPP <- get("last_plot.phylo", envir = .PlotPhyloEnv)
  h <- max(nodeHeights(tree))
  for (i in 1:tree$Nnode) {
    ii <- round((aa$CI95[i, 1] - obj$lims[1])/d * n)
    jj <- round((aa$CI95[i, 2] - obj$lims[1])/d * (n + 1))
    cols <- obj$cols[ii:jj]
    add.color.bar(leg = 0.1 * h * v[i], cols = cols, prompt = FALSE,
                  x = lastPP$xx[i + Ntip(tree)] - 0.05 * h * v[i],
                  y = lastPP$yy[i + Ntip(tree)], title = "", subtitle = "",
                  lims = NULL, lwd = lwd)
  }
}
library(ape)
library(maps)
library(phytools)
library(tree)


data<-read.csv(file.choose())
dat<-data
mapvar<-dat$var
names(mapvar)<-dat$species
tree<-read.tree(file.choose())
asr<-contMap(tree,mapvar,plot=F)
plot(setMap(asr,col=c(1,4,5,3,7,2,6)),lwd=3)
senci.contMap(setMap(asr,col=c(1,4,5,3,7,2,6)),lwd=5,min=0,max=25.2)
```

Fibrinogen Reaction Time Script

senci.contMap is a slight modification of errorbar.contMap that trims 95% CIs of ancestral state reconstructions to a sensible range, e.g. for traits bound between 0 and 1
Example of code for implementing it would be as follows (lines separated by semicolons): pasr<-contMap(tree,mapdat,plot=F,lims=c(0,1)) ; plot(setMap(pasr,invert=T)) ; senci.contMap(setMap(pasr,invert=T),mini=0,maxi=1)

```
senci.contMap<-function(obj,...){
  if (hasArg(x))
    x <- list(...)$x
  else x <- setNames(sapply(1:Ntip(obj$tree), function(x, obj) {
    ii <- which(obj$tree$edge[, 2] == x)
    ss <- names(obj$tree$maps[[ii]][length(obj$tree$maps[[ii]])])
    obj$lims[1] + as.numeric(ss)/(length(obj$cols) - 1) *
      diff(obj$lims)
  }, obj = obj), obj$tree$tip.label)
  if (hasArg(scale.by.ci))
    scale.by.ci <- list(...)$scale.by.ci
  else scale.by.ci <- TRUE
  if (hasArg(lwd))
    lwd <- list(...)$lwd
  else lwd <- 14
  tree <- obj$tree
  aa <- fastAnc(tree, x, CI = TRUE)
  if (hasArg(min))#added lines here
    for (i in 1:length(aa$CI95[,1])){ #added lines here
      aa$CI95[i,1]<-ifelse(aa$CI95[i,1]<list(...)$min,list(...)$min,aa$CI95[i,1]) #added lines here
    } #added lines here
  else aa$CI95[,1]<-aa$CI95[,1] #added lines here
  if (hasArg(max)) #added lines here
    for (i in 1:length(aa$CI95[,2])){ #added lines here
      aa$CI95[i,2]<-ifelse(aa$CI95[i,2]>list(...)$max,list(...)$max,aa$CI95[i,2]) # added lines here
    } #added lines here
  else aa$CI95[,2]<-aa$CI95[,2] #added lines here
  xlim <- range(aa$CI95)
  if (xlim[2] > obj$lims[2] || xlim[1] < obj$lims[1]) {
    cat(paste("  -----\n  The range of the contMap object, presently (",
          round(obj$lims[1], 4), ",", round(obj$lims[2], 4),
          "), should be equal to\n  or greater than the range of the CIs on ancestral states: (",
          round(xlim[1], 4), ",", round(xlim[2], 4), ").\n",
          sep = ""))
    cat(paste("  To ensure that your error bars are correctly plotted, please recompute your\n",
          "  contMap object and increase lims.\n  -----\n",
          sep = ""))
  }
  d <- diff(obj$lims)
  if (scale.by.ci) {
    v <- aa$CI95[, 2] - aa$CI95[, 1]
    v <- v/max(v)
  }
```

```
  else v <- rep(0.5, tree$Nnode)
  n <- length(obj$cols) - 1
  lastPP <- get("last_plot.phylo", envir = .PlotPhyloEnv)
  h <- max(nodeHeights(tree))
  for (i in 1:tree$Nnode) {
    ii <- round((aa$CI95[i, 1] - obj$lims[1])/d * n)
    jj <- round((aa$CI95[i, 2] - obj$lims[1])/d * (n + 1))
    cols <- obj$cols[ii:jj]
    add.color.bar(leg = 0.1 * h * v[i], cols = cols, prompt = FALSE,
                  x = lastPP$xx[i + Ntip(tree)] - 0.05 * h * v[i],
                  y = lastPP$yy[i + Ntip(tree)], title = "", subtitle = "",
                  lims = NULL, lwd = lwd)
  }
}
library(ape)
library(maps)
library(phytools)
library(tree)


data<-read.csv(file.choose())
dat<-data
mapvar<-dat$var
names(mapvar)<-dat$species
tree<-read.tree(file.choose())
asr<-contMap(tree,mapvar,plot=F)
plot(setMap(asr,col=c(6,2,7,3,5,4,1)),lwd=3) # Reverse order of colour numbers as want blue to be
high SP and red low SP
senci.contMap(setMap(asr,col=c(6,2,7,3,5,4,1)),lwd=5,min=58.3,max=1800)
```

Fibrinogen Amplitude Script

senci.contMap is a slight modification of errorbar.contMap that trims 95% CIs of ancestral state reconstructions to a sensible range, e.g. for traits bound between 0 and 1
Example of code for implementing it would be as follows (lines separated by semicolons): pasr<-contMap(tree,mapdat,plot=F,lims=c(0,1)) ; plot(setMap(pasr,invert=T)) ; senci.contMap(setMap(pasr,invert=T),mini=0,maxi=1)

```
senci.contMap<-function(obj,...){
  if (hasArg(x))
    x <- list(...)$x
  else x <- setNames(sapply(1:Ntip(obj$tree), function(x, obj) {
    ii <- which(obj$tree$edge[, 2] == x)
    ss <- names(obj$tree$maps[[ii]][length(obj$tree$maps[[ii]])])
    obj$lims[1] + as.numeric(ss)/(length(obj$cols) - 1) *
      diff(obj$lims)
  }, obj = obj), obj$tree$tip.label)
  if (hasArg(scale.by.ci))
    scale.by.ci <- list(...)$scale.by.ci
  else scale.by.ci <- TRUE
  if (hasArg(lwd))
    lwd <- list(...)$lwd
  else lwd <- 14
  tree <- obj$tree
  aa <- fastAnc(tree, x, CI = TRUE)
  if (hasArg(min))#added lines here
    for (i in 1:length(aa$CI95[,1])){ #added lines here
      aa$CI95[i,1]<-ifelse(aa$CI95[i,1]<list(...)$min,list(...)$min,aa$CI95[i,1]) #added lines here
    } #added lines here
  else aa$CI95[,1]<-aa$CI95[,1] #added lines here
  if (hasArg(max)) #added lines here
    for (i in 1:length(aa$CI95[,2])){ #added lines here
      aa$CI95[i,2]<-ifelse(aa$CI95[i,2]>list(...)$max,list(...)$max,aa$CI95[i,2]) # added lines here
    } #added lines here
  else aa$CI95[,2]<-aa$CI95[,2] #added lines here
  xlim <- range(aa$CI95)
  if (xlim[2] > obj$lims[2] || xlim[1] < obj$lims[1]) {
    cat(paste("  -----\n  The range of the contMap object, presently (",
          round(obj$lims[1], 4), ",", round(obj$lims[2], 4),
          "), should be equal to\n  or greater than the range of the CIs on ancestral states: (",
          round(xlim[1], 4), ",", round(xlim[2], 4), ").\n",
          sep = ""))
    cat(paste("  To ensure that your error bars are correctly plotted, please recompute your\n",
          "  contMap object and increase lims.\n  -----\n",
          sep = ""))
  }
  d <- diff(obj$lims)
  if (scale.by.ci) {
    v <- aa$CI95[, 2] - aa$CI95[, 1]
    v <- v/max(v)
  }
```

```
  else v <- rep(0.5, tree$Nnode)
  n <- length(obj$cols) - 1
  lastPP <- get("last_plot.phylo", envir = .PlotPhyloEnv)
  h <- max(nodeHeights(tree))
  for (i in 1:tree$Nnode) {
    ii <- round((aa$CI95[i, 1] - obj$lims[1])/d * n)
    jj <- round((aa$CI95[i, 2] - obj$lims[1])/d * (n + 1))
    cols <- obj$cols[ii:jj]
    add.color.bar(leg = 0.1 * h * v[i], cols = cols, prompt = FALSE,
                  x = lastPP$xx[i + Ntip(tree)] - 0.05 * h * v[i],
                  y = lastPP$yy[i + Ntip(tree)], title = "", subtitle = "",
                  lims = NULL, lwd = lwd)
  }
}
library(ape)
library(maps)
library(phytools)
library(tree)


data<-read.csv(file.choose())
dat<-data
mapvar<-dat$var
names(mapvar)<-dat$species
tree<-read.tree(file.choose())
asr<-contMap(tree,mapvar,plot=F)
plot(setMap(asr,col=c(1,4,5,3,7,2,6)),lwd=3)
senci.contMap(setMap(asr,col=c(1,4,5,3,7,2,6)),lwd=5,min=0,max=6.3)
```
